# Association of Iron Deficiency Anaemia With the First Episode of Febrile Seizure in Children

**DOI:** 10.7759/cureus.74129

**Published:** 2024-11-21

**Authors:** Renuka Jadhav, Vineeta Pande, Balakrushna Garud, Shailaja Mane

**Affiliations:** 1 Paediatrics, Dr. D. Y. Patil Medical College, Hospital and Research Centre, Dr. D. Y. Patil Vidyapeeth (Deemed to be University), Pune, IND

**Keywords:** febrile seizure, iron deficiency anaemia, oligodendrocyte, serum ferritin, serum tibc

## Abstract

Introduction

Febrile seizures are the most common type of seizure in neurologically healthy children under six years of age. Iron deficiency is a prevalent micronutrient deficiency worldwide, though it is medically preventable and treatable. In many developing countries, anaemia remains a significant concern in young children. Iron plays a crucial role in neurodevelopment and overall bodily growth, and low levels of serum ferritin may lower the seizure threshold.

Methods

The study aimed to determine the correlation of iron profiles between patients with their first episode of febrile seizures and patients with only febrile illnesses. This case-control study enrolled children between 6 and 60 months of age. The case group comprised children who experienced their first episode of febrile seizure, while the control group included children presenting with febrile illness without seizures. The cost of the iron profile was waived for patients enrolled in the study. The study was conducted at a tertiary care centre and charitable hospital. Total blood counts and iron profiles were analyzed to find the association between the risk of febrile seizures and anaemia.

Results

The study included 150 children, with 50 febrile seizure cases and 100 controls with only febrile illness. The mean age of cases was 2.15 years, while that of the controls was 1.73 years. Males were more prevalent in the febrile seizure group, with 31 (62%) compared to 19 (38%) females. The mean serum transferrin value in cases was 22.46, compared to 28.42 in controls, indicating lower levels in cases (p=0.029). The mean TIBC in cases was 434.84, higher than the control group's 334.46 (p<0.001). The mean serum iron level in cases was 77.64, lower than that of controls at 86.63, though the difference was not significant (p=0.195). RDW >15% was observed in 46 (92%) cases compared to 57 (57%) controls (p<0.001).

Conclusion

Iron deficiency anaemia is a correctable risk factor for children with febrile seizures between 6 and 60 months of age. Timely diagnosis and correction of anaemia may help prevent febrile seizures in these children. Further research is needed to evaluate effective prevention strategies.

## Introduction

Globally, around 40% of children aged up to five years are affected by anaemia. Anaemia is characterised by decreased haemoglobin levels according to World Health Organization (WHO) guidelines. The cutoff values for haemoglobin levels < 11 g/dL for younger children differ according to age, sex, and nutritional status [1]. Anaemia is defined as reduced red blood cells and haemoglobin in a patient, which cannot meet the body's oxygen demand [2]. Considering the global burden of anaemia and the scant literature available on it, we were prompted to conduct a more detailed study. The most frequent convulsive condition in children is a febrile seizure, affecting 2% to 5% of children between the ages of 6 and 60 months [3]. Independent risk factors for febrile seizures include a history of frequent hospital admissions, younger age (less than two years), frequent and high-grade febrile episodes, and maternal addictions such as alcohol, smoking, and drug use during the gestational period [2]. The aetiology of febrile seizures is heavily influenced by genetics, with families of affected patients exhibiting a higher risk. Mutations in genes such as GABA receptors and the sodium voltage-gated channel alpha subunit are known contributors [3]. Fever-induced febrile seizures are believed to result from an immature brain's response to fever. Common causes of fever include upper respiratory tract, urinary tract, and gastrointestinal infections. Febrile seizures are classified as simple or complex. Simple febrile seizures occur without neurological abnormalities, while complex febrile seizures are characterised by prolonged duration (more than 15 minutes) or recurrent episodes within 24 hours, which may predispose individuals to epilepsy [4-9].

The National Family Health Survey-5 reports a worldwide increase in the prevalence of iron deficiency anaemia, from 59% to 67% in children under five years of age [1]. Since febrile seizures are more prevalent in children under two years old, and iron deficiency anaemia is also common in this age group [10], recent research has indicated that iron deficiency may be a risk factor for febrile seizures [11]. Haemoglobin plays a crucial role in transporting oxygen to various tissues and organs, including the brain [11,12]. Excessive extracellular dopamine and norepinephrine, coupled with a decrease in all monoamine transporters, have been linked to iron deficiency [13]. Microtubule-associated protein 2 expression in dendritic structures is altered by iron deprivation during pregnancy. Iron plays a significant role in the myelination of the mouse brain, with potential causes including alterations in early pre-oligodendrocyte and oligodendrocyte cells and modified regulation of oligodendrocyte iron intake through transferrin and transferrin receptors [14]. Low serum ferritin levels may lower the threshold for febrile seizures [15-16]. Infants with iron deficiency anaemia often exhibit emotional alterations such as apprehension, hesitance, reduced positive affect, and lower social engagement capacity [17]. During the embryonic period, preference is given to the development of red blood cells over other structures, such as the brain. When the iron supply does not meet demand, even if the baby is not anaemic, the fetal brain may still be at risk [18].

## Materials and methods

The study aimed to determine the correlation of iron profiles between the first episode of febrile seizures and patients with febrile illness alone. The primary objective was to compare the iron profiles in case and control groups.

We conducted this case-control study at the Department of Paediatrics, Dr. D. Y. Patil Medical College, Hospital and Research Centre, Pune, over a total duration of 24 months. This was a hospital-based study. Patients were enrolled as they were admitted, employing a convenience sampling strategy. After obtaining approval from the Institutional Ethics Committee and parental consent, 150 patients were enrolled. Among these, 100 had febrile illnesses without seizures, while 50 presented with their first episode of febrile seizures.

Children aged 6 to 60 months with fever and a first episode of febrile seizures were included as cases, while children with febrile illness without seizures were enrolled as controls. Children receiving iron therapy, those with developmental delays, and those with a history of febrile seizures were excluded.

A detailed history and clinical examination were conducted on all subjects presenting with temperatures above 38°C. Haematocrit, haemoglobin, plasma ferritin, mean corpuscular haemoglobin concentration (MCHC), serum ferritin, mean corpuscular haemoglobin (MCH), total iron-binding capacity (TIBC), and mean corpuscular volume (MCV) were measured in all patients upon admission.

Statistical analysis was performed using Microsoft Excel 365 (Microsoft Corporation, Redmond, Washington) and IBM SPSS Statistics for Windows, Version 27 (Released 2020; IBM Corp., Armonk, New York). Quantitative data were represented using mean and standard deviation, and normality was tested using the Shapiro-Wilk test. As the data distribution was normal, mean differences were analysed using an independent samples t-test. Qualitative variables were summarised as frequency and percentage and analysed using the chi-square test. For all tests, p-values <0.05 were considered statistically significant.

## Results

Out of 150 children, 50 were cases of febrile seizures, while 100 were controls with only febrile illness. The mean ages of the case and control groups were 2.15 years (SD: 1.25) and 1.73 years (SD: 0.993), respectively. The age difference was statistically significant (p=0.027), indicating that the controls were younger than the cases (Table 1).

As shown in Table 1, males were more prevalent in the febrile seizure group (31 (62%)) compared to females (19 (38%)). There was no statistically significant difference in the distribution of males between the cases and controls (p=0.324). Cases had a higher body weight than controls, and this difference was statistically significant (p=0.001).

A comparison of the causes of febrile illness between cases and controls showed no significant difference (p=0.312); the most common cause of febrile illness was respiratory tract infection, which was equally prevalent (76%) in both groups.

**Table 1:** Demographic profile and clinical symptoms of patients in cases and controls Values are represented as mean ± SD for quantitative variables (test used: Independent samples t-test) and frequency (%) for qualitative variables (test used: Chi-square test). *P-value < 0.05 is considered statistically significant.

Personal Information	Cases, n = 50 (33%)	Controls, n = 100 (67%)	P-value
Age (year)	2.15 ± 1.25	1.73 ± 0.993	0.027*
Male; n (%)	31 (62%)	52 (52%)	0.324
Weight (kg)	9.98 ± 2.3	8.65 ± 2.18	0.001*
Family history of febrile seizure, n (%)	3 (6%)	2 (2%)	0.421
Body temperature (°C)	38.56 ± 0.80	38.76 ± 0.86	0.016*
Cause of fever, n (%)			
Respiratory	38 (76%)	76 (76%)	0.312
Viral fever	9 (18%)	10 (10%)	
Gastroenteritis	2 (4%)	9 (9%)	
Cause of fever, n (%)	1 (2%)	5 (5%)	

There was a significant difference (p=0.010) in the mean value of serum ferritin between cases and controls (Table 2). The mean value of serum transferrin in cases was 22.46 ± 18.45, compared to 28.42 ± 13.98 in controls, indicating lower levels in cases than in controls (p=0.029). Meanwhile, the TIBC in cases (434.84 ± 125.18) was higher than in controls (334.46 ± 103.12) (p<0.001). The mean value of serum iron in cases (77.64 ± 44.71) was lower than in controls (86.63 ± 37.22), though the difference was not statistically significant (p=0.195).

**Table 2:** Detailed information of haematological and serum iron profile of patients in cases and controls Values are represented as mean ± SD for quantitative variables (test used: Independent samples t-test) and as frequency (%) for qualitative variables (test used: Chi-square test). *P-value < 0.05 indicates statistical significance. TIBC: total iron-binding capacity.

Investigation	Cases, n = 50 (33%)	Controls, n = 100 (67%)	P-value
Haemoglobin (g/dL)	8.62 ± 1.20	9.49 ± 1.11	<0.001*
Red cell distribution width (%)	18.02 ± 2.00	16.10 ± 1.92	<0.001*
Mean corpuscular volume (fL)	69.14 ±13.30	80.29 ± 12.46	<0.001*
Mean corpuscular haemoglobin (pg)	22.36 ± 3.80	23.91 ± 4.13	0.028*
Iron deficiency anaemia, n (%)	31 (62%)	16 (16%)	<0.001*
Serum ferritin (ng/mL)	49.84 ± 65.80	80.64 ± 69.42	0.010*
Serum iron (µg/dL)	77.64 ± 44.71	86.63 ± 37.22	0.195
Serum TIBC (µg/dL)	434.84 ± 125.20	334.46 ± 103.12	<0.001*
Serum transferrin (%)	22.46 ± 18.45	28.42 ± 13.98	0.029*

In this study, we observed that 48 (96%) of cases and 85 (85%) of controls were anaemic (Hb < 11 g/dL), which was statistically significantly different (p=0.045). RDW > 15% was observed in 46 (92%) of cases, which was significantly greater than in controls, where it was 57 (57%) (p<0.001) (Table 3) [1].

**Table 3:** Association of anaemia and related markers with risk of febrile seizure Values are represented as frequency (%) (test used: Chi-square test). *P-value < 0.05 is considered statistically significant. RDW: red cell distribution width.

Haemoglobin	Cases, n = 50 (33%)	Controls, n = 100 (67%)	P-value
Anaemia (Hb < 11 g/dL)	48 (96%)	85 (85%)	0.045*
Normal (Hb ≥ 11 g/dL)	2 (4%)	15 (15%)	
RDW>15 %	46 (92%)	57 (57%)	<0.001*
RDW<15 %	4 (8%)	43 (43%)	

## Discussion

Iron deficiency anaemia is a more frequent clinical finding in children with febrile seizures compared to those with febrile illness alone. Iron deficiency anaemia is a correctable risk factor for children with febrile seizures between 6 and 60 months of age. Serum iron plays an important role in cognitive, neurotransmitter, and neurodevelopmental functions [19]. In this study, most patients (62%) were male, while in the control group, the gender split was nearly even, with 52% male and 48% female. Sharif et al. found 62% of males in the cases and 56% of patients in the control group being male [20]. Most of the cases (82%) and controls (73%) had a temperature on admission between 38.33°C and 38.89°C. In some cases, the mean maximum temperature was 38.92 ± 1.1°C, whereas in controls, it was 38.41 ± 1.1°C. Ghasemi et al. reported that 54 cases (mean temperature 38.89 ± 1°C) and controls (mean temperature 38.5 ± 1°C) had a significant temperature difference [21]. Febrile seizures were found to run in the family in 6% of cases and only 2% of the control group. A family history of febrile seizures was associated with a three-fold increase in the likelihood of developing a febrile seizure, although this association was not statistically significant. Daoud et al. and Kumari et al. also identified a strong link between febrile seizures and a family history [22,23]. In this study, seizures lasting less than 15 minutes were reported in 28 males and 16 females. The research further found that 28 males and 18 females experienced generalised tonic-clonic seizures. Only one incidence of focal seizure and three occurrences of febrile state were reported in the study by Habibian et al. [24].

Children aged 1 and 2 comprised the majority of cases and controls in this study. The average age in both cases (2.15 ± 1.25 years) and controls (1.73 ± 0.993 years) was approximately 2.15 years. In research conducted in Iran, 54% of participants in the febrile seizure group were under two years of age (17% were less than one year, 37% were between 1 and 2 years, 22% were between two and three years, and 24% were over three years) [20]. Additionally, half of the patients in the study by Bidabadi and Mashouf were under two years old, compared to fewer than half of the cases examined in studies on iron deficiency anaemia [13]. In a study by Sharif et al., 72% of patients were under the age of two years [20]. Anaemia was observed in 96% of cases and 86% of controls in this study, with the likelihood of a febrile seizure being four times higher in anaemic patients than in non-anaemic patients. Research by Ghasemi et al. found that 40% of children with febrile seizures had iron-deficiency anaemia compared to 26% of children with febrile illness without seizures and only 12% of healthy children [21].

The mean value of serum ferritin in cases was 49.84 ± 65.80, compared to 80.64 ± 69.42 in controls. Transferrin levels in cases were 22.46 ± 18.45, while controls had a mean value of 28.42 ± 13.98. Both were lower in cases compared to controls. The TIBC value in cases was 434.84 ± 125.18, whereas in controls it was 334.46 ± 103.12, indicating higher levels in cases than controls. These differences were found to be statistically significant. The mean value of serum iron in cases was 77.64 ± 44.71, compared to 86.63 ± 37.22 in controls, which was lower in cases than in controls; however, this difference was statistically non-significant. According to Sharif et al., there was no significant change in Hb, serum iron, or TIBC levels between patients and controls, with a statistically insignificant change (p>0.05) [20]. Ghasemi et al. reported a significant difference in the mean values of haematocrit (Hb), haematocrit (RCB), ferritin (S. Iron), TIBC, and transferrin saturation between patients with febrile seizures and healthy controls [21]. Fallah et al. found that children with febrile seizures had haemoglobin levels lower than those of healthy children (11.46 ± 1.18 g/dL vs. 11.9 ± 0.89 g/dL, p=0.042), serum iron levels lower than those of healthy children (75.13 ± 35.57 g/dL vs. 38.52 ± 11.38 mg/mL, p=0.001), and serum ferritin levels that indicated higher rates of iron deficiency (48% vs. 28%; odds ratio=4.3; p=0.03) and iron deficiency anaemia (22% vs. 10%; odds ratio=3.16, p=0.04) [25].

Limitations

Due to the small sample size, we could not generalise these findings to this age group. We were unable to collect health records post-discharge. In our study, we enrolled patients as cases who were developmentally normal and experiencing their first episode of febrile seizure. We were unable to follow up with them.

## Conclusions

This study highlights a significant relationship between iron deficiency anaemia and the incidence of febrile seizures in children aged 6 to 60 months. Screening for iron deficiency anaemia should be prioritised for children experiencing their first seizure episode, as early diagnosis and timely iron supplementation may help mitigate the risk. Given the high prevalence of anaemia in this age group, addressing nutritional deficiencies is crucial for promoting overall health and neurological development. Further research is needed to explore this connection and evaluate effective prevention strategies.
